# Introducing internet-based cognitive behavioral therapy in the Latvian government-funded mental health sector

**DOI:** 10.3389/fpsyt.2025.1657309

**Published:** 2025-10-08

**Authors:** Ieva Kince-Laus, Liene Sile, Jurijs Novickis, Elizabete Romanovska, Liene Dambiņa, Maris Taube

**Affiliations:** ^1^ Department of Psychosomatic Medicine and Psychotherapy, Riga Stradiņš University, Riga, Latvia; ^2^ Scientific Institute of Mental Health, National Mental Health Center, Riga, Latvia; ^3^ Child and Adolescent Resource Center, Riga, Latvia

**Keywords:** digital therapy, CBT, anxiety, depression, iCBT, psychotherapy, Latvia

## Abstract

As mental health challenges grow globally, innovative interventions are being sought. Internet-based Cognitive Behavioral Therapy (iCBT) offers a promising alternative to traditional psychotherapy—reducing costs, improving accessibility, and addressing healthcare worker shortages in the public sector—essential for Latvia, where many people live in rural areas, have limited income and there is a lack of mental health specialists, making it difficult for patients to access psychological support. In 2024, Latvia launched its first government-funded iCBT pilot. This study introduces the framework and implementation strategy of the Latvian iCBT pilot, done in collaboration with Finland’s HUS and the HealthFox platform. The program targets young adults from the age of 18 to 25 with mild to moderate depression and anxiety, based on validated clinical thresholds (PHQ-9 >8, GAD-7 >10). The population clinical symptoms were designed similarly to previous experience with iCBT evaluated in Finland. The structured therapy, delivered through a mobile app, includes weekly guided sessions, personalized therapist feedback, and interactive digital modules. This article examines the architecture of the pilot—its referral system, therapy modules, data collection process, and therapist responsibilities. Also, it is looking at it within broader global evidence on iCBT efficacy, dropout rates, and patient satisfaction.

## Introduction

The variety of web technologies and smartphones has transformed our lives in every imaginable way, including new approaches for assessing and treating psychiatric disorders. Cognitive-behavioral therapy (CBT) is traditionally delivered face-to-face through direct patient–therapist interactions. Digital CBT can be described as the use of digital tools, applications, or devices to deliver or support the assessment, formulation, treatment, training, and monitoring of CBT. CBT implementation is theoretically grounded in Beck’s cognitive model, particularly the cognitive triad, which describes the role of automatic negative thoughts about the self, the world, and the future in the development and maintenance of depression (1). In addition, CBT interventions often employ functional analysis using the ABC (Antecedent–Behavior–Consequence) model to help patients understand the links between triggers, thoughts, behaviors, and outcomes. In this approach, the ABC model serves as a practical instrument to structure the therapeutic work during CBT sessions. In practice, the therapist assists in identifying patients’ specific challenges and supports the development of coping strategies. Digital CBT typically consists of 8–12 structured modules, incorporating audio and visual materials, homework assignments, and skill-building exercises (2).

In recent times, specialists worldwide have encountered significant changes in the enforcement of digital CBT in response to the COVID-19 pandemic. During that time, CBT providers and healthcare workers needed to shift towards remote services and successfully continue executing sessions in online settings. The pandemic has ended, but the need to provide online CBT sessions remains, primarily due to individual patient needs and preferences, varying living locations, financial constraints, and a shortage of healthcare professionals. In Latvia, psychologists play an essential role in multidisciplinary teams providing mental health care across all age groups. They contribute to early diagnostics, psychological assessments, individual and group therapies, and psychoeducation, particularly within services for children, adolescents, and adults with behavioral and emotional disorders. Psychologists are also actively involved in community-based care models aimed at reducing hospitalization needs and promoting patients’ social inclusion and well-being. These priorities are outlined in the *Mental Health Care Organization Improvement Plan 2023–2025* approved by the Cabinet of Ministers of Latvia (3).

Latvia faces significant challenges in mental health care access. In 2022, Eurostat showed that Latvia has one of the lowest numbers of psychiatrists per capita in the EU—approximately 15 per 100 000, compared to the EU average of 22. Waiting times for publicly funded psychological services can exceed several months, especially outside bigger cities. Limited funding, centralization of services in urban areas, and societal stigma further reduce access to care (4). In rural regions, access is even more restricted due to transportation issues and a lack of mental health professionals. This highlights the urgent need for realistic digital solutions, such as iCBT.

In real-life practice, there are struggles with availability and access to these specialists because of their ability to consult patients in the government sector, e.g., approximately 3 patients a day from empirical hospital data analyses in adult settings. This means that individuals can seek treatment from a licensed psychiatrist without the need for an intermediary. It grants patients the freedom to take an active role in managing their healthcare and eliminates unnecessary delays in accessing therapy services. It is a state-funded service (5). Each medical institution, which provides state-funded services, has an outpatient department where state-funded psychiatrist services and consultations are provided.

The Latvian psychiatric care system is primarily state-funded and organized based on a centralized model coordinated by the National Health Service. Outpatient psychiatric consultations are available in most regions, but the scope of psychological services is much more limited, with most of them concentrated in urban centers such as Riga (3). Furthermore, state-funded adult psychologist consultations are not immediately available due to specialist shortages and increasing demand. A nationwide cross-sectional primary care study (2020) showed that the current prevalence of any mental disorder in Latvia was 37.2% and was significantly greater in women. Mood disorders (18.4%), suicidality (18.6%), and anxiety disorders (15.8%) were the most frequent diagnostic categories, it is highlighting the need for searching for more effective strategies to deliver support and psychological consultation for those in need (6).

Some studies reported results suggesting that the shift toward online therapy did not produce a worsening of therapy health outcomes (7, 8). Especially promising results of the therapy were achieved in younger patients, but elderly patients achieved fewer benefits from the remote type of therapy. In young individuals, a significant reduction in depression and anxiety symptoms was observed. The results also highlight the willingness and ability of CBT practitioners to adapt to a frequently changing environment and implement remote CBT sessions.

Studies on internet-based CBT started in the 1990s (9). The goal was to provide patients with the same content and length of the CBT program as face-to-face therapy. From that time, more than 180 randomized control trials were conducted with promising results: internet-based CBT was clinically effective compared to control groups (10). The ICBT treatment model was also successfully used for child and adolescent therapy (11).

According to the systematic reviews on the comparison of face-to-face CBT to iCBT, the latter is considered even more effective for treating social anxiety disorder (10). The possible explanation is that the therapist can be perceived as an anxiety-producing object in traditional therapy settings.

Therapeutic alliance is worth considering when comparing classical face-to-face CBT with iCBT. While therapeutic alliance is present in both settings, there is a shift in patient perception of interactions in iCBT. Compared to face-to-face CBT, when the patient interacts only with healthcare professionals, iCBT interactions include recorded materials, tasks, and self-awareness exercises (12). Opposite to previous expectations, researchers concluded that patients rated the therapeutic alliance higher in online therapy interventions compared to traditional therapy (13). Studies confirm a correlation between the therapeutic alliance score and patient health outcomes (14–16).

In December 2024, the iCBT project was launched in Latvia in collaboration with Finland’s HUS and the HealthFox digital platform. As part of Latvia’s mental health innovation efforts, the National Mental Health Centre(NMHC) is piloting a semi-automated internet-based cognitive behavioral therapy (iCBT) program. The initiative aims to expand access to psychological support by offering guided self-help interventions, enabling patients to engage in therapy with remote supervision from mental health professionals. The iCBT project represents a step toward digitalizing mental health services and integrating technology-assisted care into the national healthcare system. Standardized programs, adopted and adapted from Finland, are intended for depression, generalized anxiety disorder, and social anxiety. Three healthcare institutions in Latvia are involved in the implementation process and service delivery: the Child and Adolescent Resource Centre (CARC), the Children’s Clinical University Hospital, and the National Mental Health Centre. The target group are individuals aged 18 to 25 years, who have been diagnosed with mild or moderate depression or scored more than 8 points in Patient Health Questionnaire-9 scale (PHQ-9, 17) and/or individuals with anxiety syndrome who scored more than 10 points in General Anxiety Disorder-7 scale (GAD-7, 18). Typically, it takes between 1 to 7 days from the initial contact for a patient to be included in the program, following the algorithm steps, that include initial contact, psychiatric evaluation, document completion, creating a profile in the system, entering patient data, and connecting with the responsible psychologist.

## Methodology

The methodological standard of patient inclusion was adapted from colleagues at the Helsinki University Hospital (HUS), who have developed several iCBT protocols, including for depression (19). Their collaboration partner, HealthFox, provides the digital platform used for the delivery of these interventions in pilot projects. Thus, in Latvia, we received an adaptation-ready product that had already undergone research and validation in Finland and therefore did not require a separate ethical committee evaluation. Nevertheless, all methods followed good clinical practice. All patients received authorized and approved instructions in written form, which were additionally explained by a specialist. Before enrolling in the iCBT program, all participants provided written informed consent.

As mentioned earlier, the study includes patients of the NMHC, who have met the criteria for affective disorders and all neurotic spectrum disorders according to ICD-10 classification (20) and have scored more than 8 points in PHQ-9 scale and/or more than 10 points in GAD-7 scale. These criteria are identified by a psychiatrist or a family doctor on a first or regular appointment, who has previously received a description about iCBT via work email, including indications and contraindications referring patients, and has access to a recorded video lecture about iCBT. The patient should be able to understand the material in Latvian, have access to the internet to download the materials, and have a smart device, such as a smartphone or tablet, to use the app.

The procedure for a patient to be included in iCBT is as follows: a doctor explains to a patient, an adolescent or a young adult that digital therapy iCBT (term for the patients) is an innovative, evidence-based method that can help address mental health issues through smart devices. Before starting iCBT therapy, the patient and their legal representative must read and complete the patient’s informed consent form, which contains information about the program and that it is being implemented as a pilot project, the target audience, the specialist with whom communication is taking place, the need and scope of the service, the benefits, possible risks and alternatives to the recommended program, termination of participation in the program if the patient has not engaged in any program activity without a valid reason, confidentiality and the possibility of refusing participation in the program, after which the specialist may contact the patient or legal representative to clarify the reason for refusing participation and invite them to fill out the questionnaires. The form is signed by a patient or their legal representative and a psychiatrist or psychologist.

**Figure 1:**
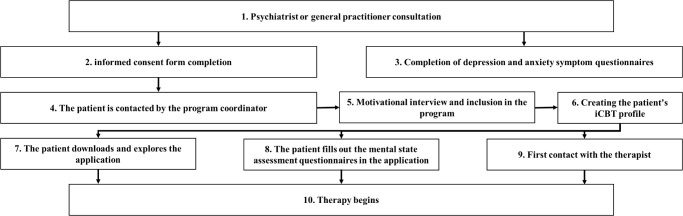
Flowchart of patient inclusion and initiation process in the internet-delivered cognitive behavioral therapy (iCBT) program, from initial psychiatric/general practitioner consultation to therapy initiation.

Information about the patient, doctor’s referral, patient consent form, PHQ-9, and GAD-7 scale results are transferred to the project coordinator, or the patient is given the coordinator’s phone number, and the patient can contact the coordinator himself. The coordinator’s tasks are to introduce the patient to the service and the content of the pilot project, verify the admission criteria, conduct an introductory interview, issue an access code to application, download the application with the patient and present the technical side, create the patient’s iCBT profile, add therapy, enter, store and update patient’s data in the system, and transfer information about the patient to iCBT therapist. The patient explores an application until the first contact with the therapist and fills out the mental state assessment questionnaires in the application. The patient’s flowchart for the iCBT therapy is seen in **Figure 1**.

iCBT therapy lasts for 2–5 months and includes weekly 1-hour sessions with a therapist, written, video and audio materials. The sessions are followed by daily exercises, such as writing a digital diary about mood and thoughts, guided audio meditations, cognitive restructuring activities, behavioral activation methods, self-assessment, and other methods that help to practically apply the learned skills in everyday life. The patient also sees progress in the form of a daily summary and overall progress. The process, protocols, and use of alternative tools are based on the clinical experience of Helsinki University Hospital (21).

Currently, the iCBT app has the Depression Module and the Generalized Anxiety Module in a test mode. The Social Anxiety module will also be introduced soon. The Depression Module consists of 7 sessions, where questionnaires and tests are used in the assessment of the mental status (**Table 1**).

**Table 1:** Assessment tools used at each session of the depression module.

	S0	S1	S2	S3	S4	S5	S6	S7
	Admission	Welcome to online depression therapy	Cognitive perspective	Thoughts and well-being	Thoughts and their impact	Values and beliefs	Comprehensive well-being	Wrapping up
PHQ-9	X	X	X	X	X	X	X	X
GAD-7	X	X		X		X		X
AUDIT-C	X	X		X				X
MSPSS	X	X		X		X		X
WHO-5	X	X						X
C-SSRS	X	X	X	X	X	X	X	X

PHQ-9, Patient Health Questionnaire-9; GAD-7, Generalized Anxiety Disorder 7-item scale; AUDIT-C, Alcohol Use Disorders Identification Test; MSPSS, Multidimensional Scale of Perceived Social Support; WHO-5, World Health Organization Well-Being Index; C-SSRS, Columbia Suicide Severity Rating Scale; S1–S7, sessions of The Depression Module.

Patient Health Questionnaire-9 (PHQ-9) is assessed during all sessions to determine the severity of depression. Generalized Anxiety Disorder-7 (GAD-7) is assessed during every other session to screen patients for the development of anxiety and to grade its severity. Alcohol Use Disorders Identification Test (AUDIT-C) (22) is assessed in Session 1, Session 3, and the last session to identify patients who are hazardous drinkers or who may have an active alcohol use disorder. Multidimensional scale of perceived social support (MSPSS) (23) is a 12-item measure of perceived adequacy of social support from three sources: family, friends, & significant other, and the patient’s assessment of it occurs in every other session. During the first and last sessions World Health Organization Well-Being Index (WHO-5) (24) is assessed to state overall mental well-being. During all sessions, self-harm risk is assessed with the Columbia-Suicide Severity Rating Scale (C-SSRS) (25). All assessment tools are used at admission and at the last session.

After the completion of Session 7, the patient is monitored for 3 months. The daily tasks of the Depression Module include an activity diary, a thought diary, a sleep diary, problem-solving methods, relaxation techniques, and situation analysis.

The Generalized Anxiety Module contains 12 sessions during which assessment tools are taken (**Table 2**). Three months after the end of Session 12, a follow-up is conducted. Tasks of the module include Saying “No” exercises, a Thought, Worry and Relaxation Diary, Imaginative Exposure and others. The mandatory tests to be completed are AUDIT-C, GAD-7, PHQ-9 and WHO-5.

**Table 2:** Assessment tools used at each session of the generalized axiety module.

	S0	S1	S2	S3	S4	S5	S6	S7	S8	S9	S10	S11	S12
	Admission	Overall overview of GAD	Relaxation and the body	Worrying or panicking	Experiences of worry times	Thoughts associated with worrying	Supposed benefits of worrying	Introduction to worry removal	Anxiety questionnaire	Introduction to accepting uncertainty	Introduction to problem solving skills	Problem solving assessment	Introduction to maintaining achievement
PHQ-9	X			X		X		X		X			X
GAD-7	X	X	X	X	X	X	X	X	X	X	X	X	X
AUDIT-C	X												X
WHO-5	X												X

PHQ-9, Patient Health Questionnaire-9; GAD-7, Generalized Anxiety Disorder 7-item scale; AUDIT-C, Alcohol Use Disorders Identification Test; WHO-5, World Health Organization Well-Being Index; S1–S12, sessions of The Generalized Anxiety Module.

Testing for the Social Anxiety Module has not yet begun, and the outline of the module is not yet clearly known. It is known that the module will consist of 7 or 8 sessions, and there is a follow-up 3 months after the end of the therapy, during which the therapist communicates with the patient.

In addition, iCBT therapy includes structured weekly remote sessions supervised by a trained therapist, and an opportunity to chat with the therapist. The therapist is a qualified psychologist who provides feedback during therapy, comments on tasks, and answers questions using safe and reliable communication. Feedback should always be individualized and personalized. It should be linked to previous tasks (referring to the patient’s progress, mentioning previous messages). It is desirable to normalize and validate the patient’s feelings, sensations, and emotions, note and be interested in the patient’s well-being and therapy experience (changes in symptoms, motivation) and be interested in what has been helpful, discuss the progress. The patient’s situation and problems need to be conceptualized. Sharing one’s experiences should be done consciously and proportionately, and call the patient as needed.

The therapist’s basic responsibilities include therapeutic support, which are supporting the patient in implementing the main therapeutic methods (e.g., keeping an activity diary, exposure exercises, thought restructuring, etc.), giving feedback on the content and performance of sessions, providing emotional security, validation, and encouragement via writing or a telephone call, promoting the patient’s motivation to continue therapy and emphasizing his/her progress, and helping to explain or deepen understanding of the content and meaning of the therapy.

The therapist is responsible for supervision and communication, making an initial contact (preferably by phone) to explain the structure of the therapy and get to know the patient. He/she must regularly monitor the patient’s progress (every week) and respond to messages within 48 hours, contact patients who have not started or are not progressing (call, send messages in the application or SMS), stop the therapy for patients who have not progressed for at least one month (and do not respond to messages, calls) or refuse iCBT therapy. There are non waiting time availability to access an adult psychiatrist if the psychologist decides that it is needed for the patient because of his mental state deterioration.

The therapist also has administrative responsibilities, such as initial contact, final summaries and timely notification of planned vacation or break, in order to hire another therapist if necessary. A very important responsibility of a therapist is crisis management, to immediately check for suicide risk signals and act in accordance with the organization’s protocol. If any of the patients fall into a high-risk group or their condition worsens during therapy, the psychologist contacts the project psychiatrist or the patient’s psychiatrist and addresses the situation according to individual needs. The therapist must maintain professionalism and cooperation, i.e., follow the structured protocol, while individualizing communication, and regularly consult with colleagues.

After completing iCBT, the psychologist gives feedback to the treating psychiatrist, and then the experience and results of therapy are discussed in the next face-to-face consultation. If a patient has decided to drop out, the same feedback and after evaluation in a face-to-face consultation is applied.

## Results

Over a five-month period (from 01.12.2024 to 30.04.2025), a total of 120 patients were included in the iCBT program in the NMHC. Of these, 89 were enrolled in depression therapy modules and 31 in the generalized anxiety treatment program. 17 patients have successfully completed the full course of the program.

A total of 102 patients voluntarily completed the demographic data survey. Among them, 86 identified themselves as female, 15 as male, and one preferred not to disclose gender. The average age was 20.96 years. In terms of geographic distribution, 73 respondents resided in Riga (the capital city), 23 in other cities, and 6 in rural areas.

29 respondents had completed only basic education, 40 had finished secondary education, 17 had completed vocational education, 13 held a higher degree, and 1 had a master’s degree. At the time of the survey, 32 patients were university students, 25 were employed, and 16 were neither studying nor working.

71 respondents stated that at least one of their parents had completed higher education. Living arrangements varied: 47 respondents lived with parents, 22 with a friend or unregistered partner, 4 with a spouse, and 18 lived alone.

Self-assessed income levels were reported as follows: two patients classified their income as high, 19 as above average, 71 as average, eight as below average, and two as low. At the time of the survey, 75 patients were receiving medication treatment as of the moment of survey, while for 27 patients, the iCBT program constituted the primary mode of therapy.

Analyzing the factors of patients not completing the therapy, there was a trend that patients had already received the needed support during the number of sessions completed. The authors will continue with symptom analyses in the future. Right now, we confirm the trend that when patients get better in the anxiety and depression scale, they voluntarily exit the therapy, keeping the educational materials for rereading purposes.

The one thing we can learn from drop-outs is that the iCBT program is user-friendly, because the time participants decide to exit iCBT without finishing the full module was the time they got better, not the beginning, because of technical or unsatisfactory reasons with the application.

## Discussion

One of the most significant strengths of iCBT is its ability to overcome traditional barriers in mental health care. Rural populations, people with mobility limitations, and those facing financial constraints can access therapy without the need for travel or time off work (26). In the Latvian context, the introduction of iCBT represents an important step toward addressing long-standing gaps in mental health services. The program provides flexibility, allowing users to engage with therapeutic content at their own pace and on schedules that accommodate work, family, and other responsibilities. Users can revisit materials and practice interventions repeatedly, reinforcing learning and skill development (27).

Mental health services in Latvia have traditionally been centralized, with long waiting times and a shortage of psychotherapists, particularly in rural areas. iCBT allows structured CBT interventions to reach patients across the country without requiring additional infrastructure or travel. Engagement from both urban and rural patients suggests that iCBT can reduce regional inequalities in access. Furthermore, because therapists spend less time per patient compared to face-to-face treatment, iCBT has the potential to alleviate workforce shortages within the public system. The efficacy of iCBT has been demonstrated for depression, anxiety, and PTSD (28), and meta-analyses show that guided programs can lead to significant, long-term improvements in mental health symptoms (29). Patients generally report high satisfaction, particularly when programs incorporate interactive elements and therapist feedback. Although full cost-effectiveness analyses are still pending, early results suggest that iCBT may be a feasible approach to expanding evidence-based psychological support in a resource-constrained national healthcare system like Latvia.

During this project, we conducted a parallel practical evaluation of how iCBT integrates with existing psychological consultation services. While feasibility assessment was not the primary objective, the project provided insights for future implementation. This evaluation considered service costs, patient waiting times, specialist workload, and satisfaction among psychologists, psychiatrists, and patients. Overall, real-world observations in Latvia closely mirrored findings from countries with established iCBT programs, such as Finland.

From a healthcare perspective, iCBT is a cost-efficient alternative to traditional therapy, reducing clinician time, infrastructure needs, and patient transportation (30). In Latvia, where access to mental health specialists is limited and patients often must travel long distances to urban centers, these savings are particularly relevant. Research indicates that face-to-face therapy requires approximately 7.8 times more therapist time than iCBT (31). Automated and self-guided programs deliver treatment at a fraction of the cost of conventional psychotherapy, supporting financial sustainability within the Latvian public healthcare system.

iCBT can also reduce stigma-related barriers to care. Its anonymity encourages help-seeking behavior and allows patients to access treatment discreetly (32), potentially increasing engagement and reducing the societal burden of mental illness.

However, iCBT is not without challenges. One major issue is low adherence and high dropout rates. Online therapy programs generally show higher dropout than traditional therapy (33). Lack of in-person accountability can reduce motivation and commitment (34). In unguided programs, where there is minimal therapist contact, users may struggle with motivation, face technical issues, or find the program unsuitable. Without regular therapist support, patients may not feel responsible for continuing therapy or may not know how to manage difficult emotions (35). These challenges are particularly relevant in the Latvian pilot, where participants are engaging with iCBT for the first time.

Another concern is the risk of adverse effects, including symptom worsening. Patients may experience increased anxiety during exposure exercises or feel isolated while working on sensitive topics. Gullickson et al. (36) reported negative emotional effects in 16% of cases, including anxiety, frustration, hopelessness, stress, and discomfort. In traditional therapy, clinicians can detect and address these issues during sessions, whereas in iCBT, especially unsupervised programs, these effects may go unnoticed.

Lack of personalization is also a concern. Many iCBT programs are structured with prewritten modules and automated feedback, which may not be suitable for all patients. Those with complex or comorbid conditions, such as severe depression, personality disorders, or PTSD, may find these programs insufficient (27).

Risk management in crisis situations is particularly important. Standard iCBT programs do not automatically detect or respond to suicidal ideation or severe mental health episodes. In traditional therapy, clinicians can intervene, but in online settings, patients must report issues themselves. While emergency contact information may be provided, it is not always utilized. For the Latvian pilot, which serves young adults with mild to moderate depression and anxiety, therapists must monitor progress closely and implement safety protocols. This limitation makes iCBT less suitable for high-risk or unstable patients.

Digital inequality also affects accessibility. Not all patients have adequate internet access, devices, or digital literacy. Older adults, individuals with cognitive limitations, and those with lower incomes may face difficulties. Cultural and language barriers can also reduce the effectiveness of standardized iCBT programs. According to the 2023 Digital Economy and Society Index, Latvia has moderate digital skills, slightly below the EU average (45.3% *vs*. 55.6%). While internet access is high (>90% of households), gaps remain among older and low-income populations, indicating that iCBT is more feasible for younger, urban users.

Data privacy and confidentiality are additional concerns. Cyberattacks and inconsistent data storage practices create potential legal and ethical challenges. iCBT platforms store sensitive mental health information, and patients must trust that their records are secure. Legal protections for digital health data vary across countries, including Latvia.

Finally, many patients miss the human connection in iCBT. A therapeutic alliance is crucial for successful psychotherapy, and lack of face-to-face interaction may weaken emotional connection (37). Asynchronous communication or text-based interactions reduce nonverbal cues, making trust and rapport harder to establish. Guided iCBT with therapist support by email or chat helps, but some users still feel isolated.

Findings from our pilot project suggest that, for some patients, direct contact and communication with therapists represent a vital component of the therapeutic process. Typically, calls take place once per week and last around 20 minutes. Many participants reported valuing these scheduled therapy calls, with several expressing a desire for longer conversations. These interactions were often described as meaningful and supportive, contributing positively to the overall treatment experience.

## Limitations

The current study is based on preliminary, uncontrolled data, which limits the generalizability of our findings. The patient cohort is relatively small, and future work should aim to include a larger and more diverse sample to strengthen the robustness of the results. Additionally, this paper does not include a detailed evaluation of feasibility; such an assessment, including qualitative interviews with psychologists who deliver the semi-automatized digital therapy, is planned for the next phase of the project.

One of the limitations is that the intervention design did not follow a formal user-centered design (UCD-11) framework. Although the program was adapted from validated iCBT protocols and aligned with clinical practice, in this pilot project, usability was not formally assessed using standardized instruments. Similarly, acceptability was not directly evaluated through qualitative methods such as focus groups or interviews, and dropout rates can only be interpreted as a far-outcome indicator rather than a comprehensive measure of user experience.

Another limitation of the present study is the categorical diagnostic framework used, based on the convenience principle. While this approach reflects current clinical standards (ICD-10, DSM-5) and facilitates comparability with prior research, it does not fully capture the dimensional approach of psychiatric symptoms. Dimensional models, such as the RDoC framework and transdiagnostic perspectives, may provide a more nuanced understanding of heterogeneity and comorbidity. Future studies should therefore consider integrating both categorical and dimensional approaches to enhance the ecological validity and generalizability of findings.

At this stage, it is not possible to provide precise estimates of population coverage or cost-effectiveness. However, if the iCBT service is adopted as a standard practice within government-funded mental health services, future research will be able to evaluate both economic and public health impacts more rigorously. These next steps will also allow a deeper exploration of factors such as patient engagement, drop-out rates, and integration into existing mental health care pathways.

Overall, while our findings provide an initial insight into the potential of iCBT in the Latvian context, they should be interpreted cautiously, and further research will be essential to confirm these preliminary observations.

## Conclusion

We can see the growing demand from society for psychological consultations; nevertheless, the government health budget has its limitations. This approach, so far, appears promising for increasing access to psychotherapeutic methods in the government-funded mental health sector. First results in adult mental health care showed that there is space for digital technologies in psychotherapy, which are specialist-supervised, there are more gains for patients in therapy accessibility, as well as in financial efficacy, as disadvantages psychologists reported lack of face-to-face consultations; however, we agree that it should be evaluated in a controlled study.

## Data Availability

The original contributions presented in the study are included in the article/supplementary material. Further inquiries can be directed to the corresponding author.
